# *aac(6’)-Iaq*, a novel aminoglycoside acetyltransferase gene identified from an animal isolate *Brucella intermedia* DW0551

**DOI:** 10.3389/fcimb.2025.1551240

**Published:** 2025-03-11

**Authors:** Naru Lin, Wanna Xu, Dawei Huang, Chaoqun Liu, Junwan Lu, Mei Zhu, Qiyu Bao, Wei Pan

**Affiliations:** ^1^ Division of Tuberculosis Control and Prevention, Fujian Provincial Center for Disease Control and Prevention, Fuzhou, China; ^2^ Key Laboratory of Medical Genetics of Zhejiang Province, Key Laboratory of Laboratory Medicine, Ministry of Education, School of Laboratory Medicine and Life Sciences, Wenzhou Medical University, Wenzhou, China; ^3^ Department of Rehabilitation Medicine, Army 73rd Group Military Hospital, Xiamen, China; ^4^ Department of Clinical Laboratory, the People's Hospital of Yuhuan, Taizhou, Zhejiang, China; ^5^ Medical Molecular Biology Laboratory, School of Medicine, Jinhua Polytechnic, Jinhua, China; ^6^ Department of Clinical Laboratory, Zhejiang Hospital, Hangzhou, China

**Keywords:** *Brucella intermedia*, resistance mechanism, aminoglycoside acetyltransferase, *aac(6’)-Iaq*, kinetic parameter

## Abstract

**Background:**

Bacterial resistance to aminoglycoside antimicrobials is becoming increasingly severe due to their use as commonly prescribed antibiotics. The discovery of new molecular mechanisms of aminoglycoside resistance is critical for the effective treatment of bacterial infections.

**Methods:**

Bacteria in goose feces were isolated by plate streaking. The identification and characterization of a novel resistance gene from the bacterial genome involved various techniques, including molecular cloning, drug susceptibility testing, protein expression and purification, and enzyme kinetic analysis. Additionally, whole-genome sequencing and phylogenetic studies were performed.

**Results:**

*Brucella intermedia* DW0551, isolated from goose feces, was resistant to 35 antibiotics, and the minimum inhibitory concentration (MIC) was particularly high for most aminoglycoside antibiotics. The novel aminoglycoside resistance gene *aac(6’)-Iaq* encoded by *B. intermedia* DW0551 conferred resistance to netilmicin, sisomicin, amikacin, kanamycin, gentamicin, tobramycin, and ribostamycin. The amino acid sequence of AAC(6’)-Iaq shared the highest identity (52.63%) with the functionally characterized aminoglycoside acetyltransferase AAC(6’)-If. AAC(6’)-Iaq contained all the conserved sites of the acetyltransferase family NAT_SF. The enzyme exhibited strong affinity and catalytic activity toward netilmicin and sisomicin. The mobile genetic element (MGE) was not found in the flanking regions of the *aac(6’)-Iaq* and *aac(6’)-Iaq*-like genes.

**Conclusion:**

In this work, a novel aminoglycoside acetyltransferase gene, designated *aac(6’)-Iaq*, which conferred resistance to a variety of aminoglycoside antimicrobials, was identified in an animal *Brucella intermedia* isolate. Identification of new antibiotic resistance mechanisms in bacteria isolated from animals could aid in the treatment of animal and human infectious diseases caused by related bacterial species.

## Introduction

Aminoglycosides are antimicrobial molecules that are widely used in clinical practice and are usually used in combination with β-lactam drugs for the treatment of infections caused by Gram-positive and Gram-negative bacteria (Abdul-Aziz et al., 2020). Aminoglycoside resistance genes confer drug resistance to bacteria, encoding mainly aminoglycoside resistance proteins, namely, aminoglycoside acetyltransferases (AACs), aminoglycoside nucleotidyltransferases (ANTs) and aminoglycoside phosphotransferases (APHs), which acetylate, adenylate and phosphorylate drugs at specific sites, respectively (Ramirez and Tolmasky, 2010). The AAC(6’) enzyme, the most common aminoglycoside N-acetyltransferase, which modifies the 6’ site of aminoglycosides, such as kanamycin, tobramycin, gentamicin and amikacin, and usually confers resistance to these drugs (Ramirez and Tolmasky, 2010).

To date, 68 genes of the *aac(6’)* gene family have been included in the Comprehensive Antibiotic Resistance Database (CARD) (Alcock et al., 2023). These genes are located on chromosomal and plasmid DNA and are generally related to mobile genetic elements (MGEs), such as transposons and integrons (Alcock et al., 2023). Proteins encoded by the *aac(6’)* genes usually exhibit resistance to more than one aminoglycoside antibiotic. For example, AAC(6’)-Ia shows resistance to tobramycin, gentamicin, amikacin, isepamicin and netilmicin (Shaw et al., 1992), while AAC(6’)-Iy shows resistance to tobramycin, amikacin and netilmicin (Magnet et al., 1999).

*Brucella intermedia* (formerly known as *Ochrobactrum intermedium*, type strain LMG 3301 = NCTC 12171 = CNS 2-75) was first isolated from human blood in 1988 (Holmes et al., 1988) and was first described and named in 1998 (Velasco et al., 1998). These bacteria are Gram-negative facultative coccobacilli and are usually present as circular, low-convexity and smooth colonies approximately 1 mm in diameter. These bacteria generally have two chromosomes, with the larger one being responsible for activities related to functions of bacterial life and the smaller one encoding genes related to virulence. *Brucella* spp. were once thought to cause disease only in immunosuppressed populations, but more recent studies have indicated their role as opportunistic pathogens causing a wide range of diseases (Ryan and Pembroke, 2020). For example, they can lead to bacteremia in individuals with malignant tumors (Apisarnthanarak et al., 2005; Kassab et al., 2021), inflammation of the respiratory and cardiovascular systems (Hirai et al., 2016; Bharucha et al., 2017), and endophthalmitis in otherwise healthy individuals (Jacobs et al., 2013). These infections have also been reported to be associated with outbreaks of nosocomial *P. aeruginosa* infections (Sekiguchi et al., 2007). The emergence of multidrug-resistant *B. intermedia* has attracted increasing attention in recent years due to the increase in its pathogenicity (Ryan and Pembroke, 2020; Sheng et al., 2023). However, the mechanism underlying the resistance to aminoglycosides has rarely been reported (Lu et al., 2021).

In one of our recent projects, to investigate the antimicrobial resistance mechanisms of animal, human and environmental bacteria, we isolated hundreds of bacteria from fecal samples. The antimicrobial resistance profiles of these strains were examined, and their genomes were sequenced. In this work, the resistance phenotype and genotype of a microbe, designated *B. intermedia* DW0551 isolated from a goose, were characterized, and a novel chromosomal aminoglycoside resistance gene designated *aac(6’)-Iaq* was identified from this bacterium.

## Materials and methods

### Bacterial strains and plasmids

A total of 576 strains of bacteria were isolated from samples collected from sewage and domestic fowl and livestock in animal farms in Wenzhou, Zhejiang Province, China. For all of them, the minimum inhibitory concentrations (MICs) of numerous antimicrobial agents were tested, and the genomes were sequenced. The isolates were initially identified at the species level using 16S rRNA gene homology and average nucleotide identity (ANI) analyses (Konstantinidis and Tiedje, 2005). One of them, designated *B. intermedia* DW0551 herein, was isolated from goose feces. **Table 1** lists the strains and plasmids used in this study.

**Table 1:** Strains and plasmids used in this work.

Strains or plasmids	Functions	Reference
Strains		
DW0551	The wild-type strain of *Brucella intermedia* DW0551	This study
DH5α	*E. coli* DH5α was used as the host bacterium for cloning of the *aac(6’)-Iaq* gene with its upstream promoter region	CGMCC^*^
ATCC 27853	*Pseudomonas aeruginosa* ATCC 27853 was used as a quality control strain for antimicrobial susceptibility testing	CGMCC^*^
DH5α(pMD19-T-*aac(6’)-Iaq*)	*E. coli* DH5α carrying the recombinant plasmid pMD19-T-*aac(6’)-Iaq*	This study
DH5α(pMD19-T)	*E. coli* DH5α carrying the pMD19-T vector was used as a control strain for the recombinant strain in the drug susceptibility testing	CGMCC^*^
BL21(pCold I-*aac(6’)-Iaq*)	*E.coli* BL21 carrying the recombinant plasmid pCold I -*aac(6’)-Iaq*	This study
Plasmids		
pMD19-T vector	T-Vector pMD™19 (Simple) was used as a vector for cloning of the *aac(6’)-Iaq* gene with its upstream promoter region, AMP^r^	CGMCC^*^
pCold I	pCold I was used as a vector for expression of the ORF of the *aac(6′)-IVa* gene, AMP^r^	CGMCC^*^

CGMCC, China General Microbiological Culture Collection Center.

### Drug susceptibility testing

According to the M100 performance standards for antimicrobial susceptibility testing by the Clinical and Laboratory Standards Institute (CLSI) (Lewis, 2022), the MICs were determined by the agar dilution method using Mueller–Hinton (MH) agar plates with twofold serial dilutions of antibiotics (Crump et al., 2015). After 16-20 h of incubation at 37°C, the MIC results were interpreted according to M100 performance standards and the guidelines of the European Committee on Antimicrobial Susceptibility Testing (v12.0) (EUCAST, 2022). A total of 43 antimicrobial molecules were tested (**Table 2**), all of which were purchased from hospitals or pharmaceutical companies. *Pseudomonas aeruginosa* ATCC27853, also a Gram-negative bacillus as *B. intermedia* DW0551, was used for quality control. The MIC results of the quality control strain remained within the predetermined limits, indicating the credible test results.

**Table 2:** Antibiotic resistance of the strains used in this study (MIC, µg/mL).

Class	Antibiotic	DW0551	DH5α(pMD19-T-*aac(6’)-Iaq*)	DH5α(pMD19-T)	DH5α	ATCC27853
Aminoglycosides	streptomycin	1,024	2	2	2	4
	ribostamycin	4,096	256	2	≤ 1	1024
	gentamicin	≥ 512	2	0.25	0.25	0.5
	tobramycin	≥ 512	16	0.5	0.5	1
	kanamycin	≥ 4,096	8	1	1	512
	sisomicin	≥ 512	8	0.125	0.25	0.0625
	amikacin	512	8	1	1	2
	netilmicin	≥ 64	4	0.0625	0.0625	1
	paromomycin	128	4	8	4	512
	neomycin	16	2	8	16	8
Aminocyclitols	spectinomycin	512	8	8	8	64
β-Lactams	amoxicillin	256	/	/	/	1,024
	piracillin	≥ 256	≥ 256	≥ 256	8	8
	penicillin G	≥ 4,096	/	/	/	2,048
	ampicillin	1,024	/	/	/	512
	cefthiophene	1,024	/	/	/	≥ 2,048
	cefuroxime	512	/	/	/	512
	cefazolin	256	/	/	/	≥ 2,048
	ceftriaxone	≥ 256	0.0625	0.125	0.0625	32
	cefepime	≥ 32	0.0625	0.0625	0.016	0.5
	cefoxitin	256	/	/	/	≥ 1,024
	cefotaxime	256	/	/	/	8
	ceftazidime	≥ 2,048	/	/	/	1
	aztreonam	≥ 1,024	/	/	/	2
	imipenem	1	/	/	/	2
	meropenem	2	/	/	/	0.5
Quinolones	enrofloxacin	≤ 1	/	/	/	/
	levofloxacin	0.5	≤ 0.025	≤ 0.025	≤ 0.0125	0.5
	nalidixic acid	128	/	/	/	128
Tetracycline	tetracycline	16	/	/	/	4
	doxycycline	≥ 128	/	/	/	/
	tigecycline	0.5	/	/	/	0.5
	oxytetracycline	64	/	/	/	/
Chloramphenicol	chloramphenicol	128	16	16	16	128
	florfenicol	128	/	/	/	64
Macrolides	avermectin	128	/	/	/	/
	tylosin	≥ 2,048	/	/	/	/
	acetylmequine	≥ 2,048	/	/	/	/
	azithromycin	≤ 0.5	≤ 0.5	≤ 0.5	≤ 0.5	16
	erythromycin	8	/	/	/	128
Lincosamide	lincomycin	≥ 2,048	/	/	/	/
Sulfanilamide	8	/	/	/	/
fosfomycin		≥ 512	/	/	/	8

“/” the test was not performed.

### Genome sequencing, assembly, annotation, and bioinformatics analysis

The genomic DNA of the bacterium was extracted using the Universal Genomic DNA Purification Mini Spin Kit (Beyotime Biotechnology Co., Ltd., Shanghai, China). For analysis of the resistance genes in 576 bacteria, the genome of each bacterium was sequenced on the Illumina NovaSeq 6000 platform (Shanghai Personal Biotechnology Co., Ltd., Shanghai, China). The Illumina short reads of all 576 bacterial genomes were pooled together and assembled by SKESA v2.4.0 (Souvorov et al., 2018). The antibiotic resistance-related genes were annotated by Prokka (v.1.14.6) (Seemann, 2014) against the CARD (Alcock et al., 2020) and ResFinder database (Bortolaia et al., 2020). For whole-genome sequencing of *Brucella intermedia* DW0551, the PacBio Sequel II (Shanghai Personal Biotechnology Co., Ltd., Shanghai, China) sequencing platform was subsequently used. The long reads from PacBio Sequel II and the short reads from Illumina NovaSeq 6000 were hybrid assembled in a short-read-first manner using Unicycler (v0.4.8) (Wick et al., 2017) and then polished by Pilon (v 1.23) (Walker et al., 2014). The optimization of the assembly results was performed using Racon (v1.4.13) (Huang et al., 2022) and Pilon (v1.23) (Walker et al., 2014) and was assessed using QUAST (v5.0.2-fb0b821) (Gurevich et al., 2013). Putative proteins were annotated against the NCBI nonredundant protein database using DIAMOND (v2.0.14) (Buchfink et al., 2021). The average nucleotide identity (ANI) was calculated with FastANI (Zong, 2020). Multiple sequence alignment was performed with MAFFT (v7.407) (Katoh and Standley, 2013). IQ-TREE v2.2.2.3 was used to select the model that minimized the BIC score to construct a phylogenetic tree using the log-likelihood method with the Bayesian information criterion (BIC) (Lorah and Womack, 2019). The protein domains of AAC(6’)-Iaq were predicted using CD-search (Marchler-Bauer and Bryant, 2004). Genetic context analysis of *aac(6’)-Iaq* and other related sequences was carried out using clinker (v.0.0.25) (Gilchrist and Chooi, 2021).

### Cloning of the *aac(6’)-Iaq* gene

The ORF of *aac(6’)-Iaq* (447 bp) with its upstream promoter region predicted by BPROM (www.softberry.com) (**Supplementary Figure S1**) was amplified by PCR with primers designed by SnapGene v6.0 (www.snapgene.com) (**Supplementary Table S1**). The PCR products (575 bp) were subsequently ligated into the T-Vector pMD™19 using T4 ligase (Takara Biomedical Technology Co., Ltd.). The recombinant plasmid pMD19-T-*aac(6’)-Iaq* was subsequently transformed into competent *E. coli* DH5α cells, after which the recombinant strain DH5α(pMD19-T-*aac(6’)-Iaq*) was selected on LB agar plates supplemented with 100 µg/mL ampicillin. Finally, the cloned fragment [*aac(6’)-Iaq* with its upstream promoter region] was verified by Sanger sequencing (Shanghai Sunny Biotechnology Co., Ltd., Shanghai, China).

### Expression and purification of *aac(6’)-Iaq*

The PCR-amplified ORF of the *aac(6’)-Iaq* gene was inserted into the pCold I vector (**Table 1** and **Supplementary Table S1**), and the recombinant plasmid pCold I-*aac(6’)-Iaq* was subsequently transformed into *E. coli* BL21. The recombinant BL21(pColdI-*aac(6’)-Iaq*) was cultured in 100 mL of LB to an OD_600_ of 0.6 in a shaker at a constant temperature of 37°C. After cooling, 1 mM isopropyl-β-D-thiogalactopyranoside was added, and the mixture was shaken at a constant temperature of 16°C for 18 h. Bacteria were then collected by centrifugation at 10,000 × g for 10 min at 4°C and lysed with 4 mL of nondenaturing lysis solution. Subsequently, the lysed bacteria were subjected to 10 min of ultrasonic lysis. After 30 min of centrifugation (10,000 × g) at 4°C, the supernatant containing the recombinant protein was obtained, and the protein was purified using the Beyotime His-tag Protein Purification Kit (Beyotime Biotechnology Co., Ltd., Shanghai, China). The His tag of the recombinant protein was excised using Thrombin (Beijing Solarbio Science & Technology Co., Ltd., Beijing, China) at 4°C for 72 h. The AAC(6’)-Iaq protein was then concentrated using an Amicon Ultra15 centrifugal filter equipped with an Ultracel-10 membrane (Casanovas et al., 2017) and stored at -20°C with 50% glycerol.

### Enzyme kinetic analysis

The kinetic parameters of AAC(6’)-Iaq were measured as reported previously with slight modifications (Vetting et al., 2008). The AAC(6’)-Iaq activity was determined by measuring the NTB ions produced from the interaction of the acetylation reaction product CoA-SH (containing sulfhydryl groups) with DTNB (CoA-SH + DTNB → TNB^-^). The total reaction volume was 200 µL, and the pH ranged from 7-7.5. The mixture contained 2 mM DTNB, 1 mM EDTA, 100 mM CoA-SH, 25 mM MES, 5-500 µg/mL aminoglycoside substrate dissolved using sterile double-distilled water and 2 µg of purified AAC(6’)-Iaq protein (Magnet et al., 2001). The reaction was monitored at 412 nm using Synergy Neo2 (BioTek Instruments Inc., VT, United States) at a constant temperature of 37°C for 10 min at 4-second intervals. The AAC(6’)-Iaq protein in the reaction system was replaced with an equal volume of double distilled water as a control for each assay, and the experiments were repeated three times for each aminoglycoside substrate. The steady-state kinetic parameters (*k*_cat_ and *K*_m_) were determined by nonlinear regression of the initial reaction rates with the Michaelis-Menten equation in Prism (v9.4.0) software (GraphPad Software, CA, United States). Another AAC(6’) variant [AAC(6′)-Va] was analyzed together as a positive control (Zhang et al., 2023).

### Gene expression analysis by RT-qPCR

Reverse transcription quantitative PCR (RT-qPCR) was performed according to previous methods with slight changes (Rocha et al., 2020). Bacteria were cultured in LB broth until the OD_600_ reached 0.5, and for the experimental group, 1/4 MIC ribostamycin (1,024 μg/mL) was then added. The bacteria were further incubated for 2, 4, or 8 h, followed by RNA extraction with RNAprep Pure Cell/Bacteria Kit (Tiangen, Beijing, China) and quantification with a DS-11+ Spectrophotometer (DeNovix, Delaware, United States). The DNA-free RNA extract was verified by PCR of the *Brucella intermedia* 16S rRNA gene. cDNA was synthesized for each sample using HiScript III RT SuperMix for qPCR (Vazyme Biotech, Nanjing, China). RT-qPCR was performed on a CFX96™ Touch Real-Time PCR Detection System (Bio-Rad Laboratories, Hercules, CA, United States), and the increase in real-time fluorescence was monitored by using SYBR qPCR Master Mix (Vazyme Biotech, Nanjing, China). The housekeeping 16S rRNA gene was utilized as a reference gene for relevant quantification via the CT method (Schmittgen and Livak, 2008). Comparisons of expression levels were performed using a *t*-test to evaluate the effects of aminoglycosides, and p ≤ 0.05 was considered to indicate statistical significance.

### Data availability

The nucleotide sequences of the *B. intermedia* DW0551 genome have been submitted to GenBank under accession number CP131474 for chromosome_1, CP131475 for chromosome_2, CP131476 for pDW0551, and OR395485 for the *aac(6’)-Iaq* gene.

## Results and discussion

### Screening the candidate novel resistance genes

As mentioned above, to explore the resistance mechanisms of the bacteria against antimicrobial molecules, we sequenced 576 bacteria isolated from sewage, domestic fowl and livestock feces. Annotation of the pooled genomic sequences of these bacteria via Illumina sequencing demonstrated that they encoded putative resistance genes against various classes of antimicrobial agents, including resistance genes for aminoglycosides, β-lactams, fluoroquinolones, tetracyclines, phenicols, and glycopeptides. In this work, we focused on identifying new resistance mechanisms for aminoglycosides. Of the potential aminoglycoside antibiotic resistance genes annotated from the pooled genomic sequences of 576 bacteria, ten [*aph(3’)-Ia-*, *ant(9)-Ia-*, *aadA5-*, *aph(6)-Ic-*, *aph(6)-Ic-*, *aac(3)-IIIb-*, *aac(6’)-If-*, *aac(6’)-Iaa-*, *aph(6)-Id-*, and *aac(2’)-IIb-*like genes sharing < 80% amino acid sequence identity with the functionally characterized aminoglycoside resistance genes] were cloned, and their resistance functions were determined by MIC tests of several aminoglycoside antimicrobials. Finally, we found that one of these genes, an *aac(6’)-If* homolog [eventually designated *aac(6’)-Iaq* in this work] carried by the isolate DW0551 isolated from a goose, conferred resistance to several aminoglycosides.

### General features of the DW0551 genome and resistance characteristics of DW0551

**Figure 1:**
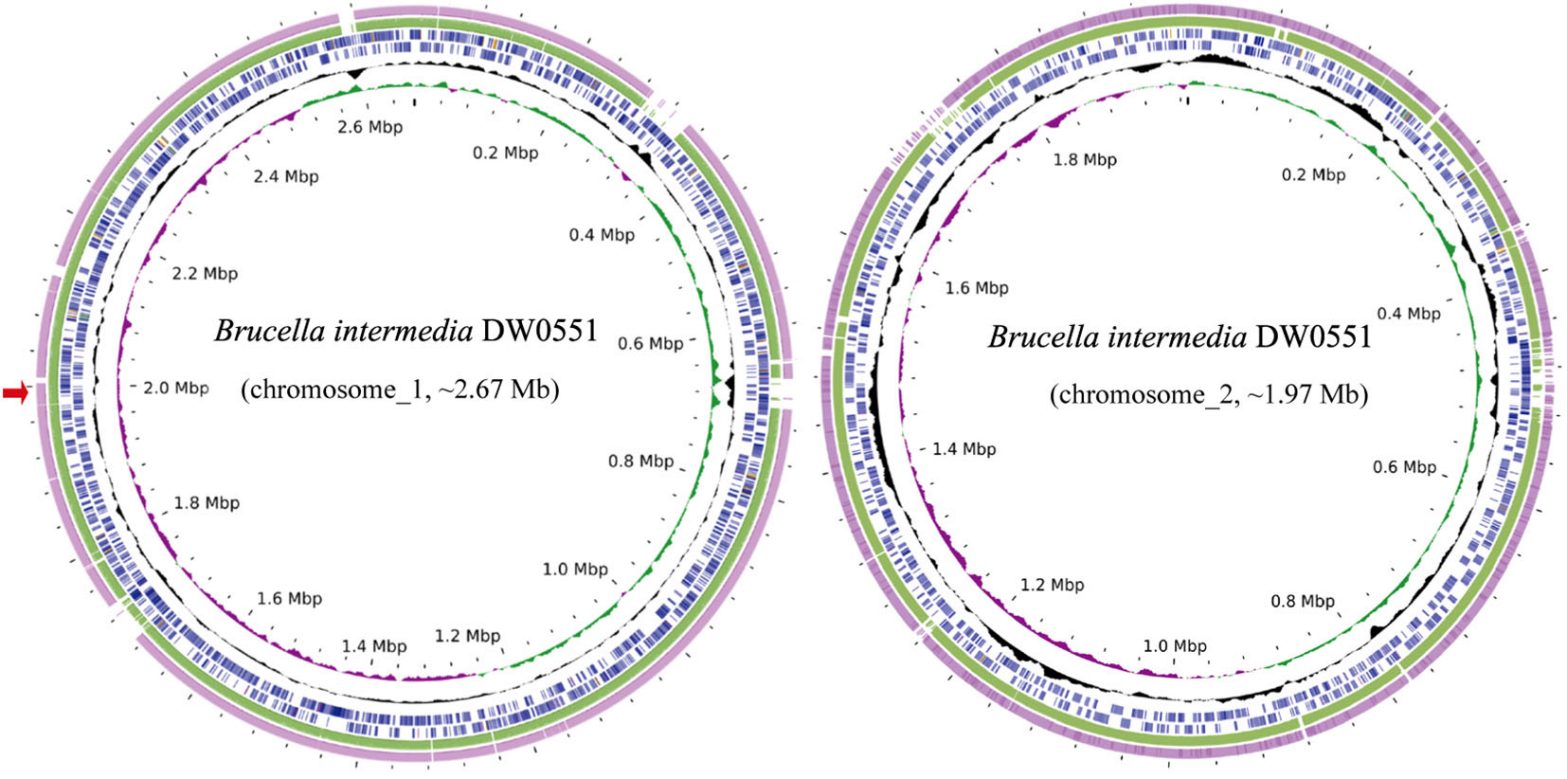
Genome maps of *B intermedia* DW0551 and its closest relatives. The circles, from inside to outside, represent the GC skew, GC content, and genes encoded in the forward and reverse strands of chromosome_1 (chromosome_2) of *B intermedia* DW0551, *B intermedia* ZJ499 chromosome 1 (chromosome 2) (CP061039.1), and *B intermedia* SG. G2 chromosome 1 (chromosome 2) (CP106662.1). The red arrow on the left represents the location of the novel resistance gene *aac(6’)-Iaq*.

To analyze the structure of the novel resistance gene-related sequence, the whole genome of DW0551, which consists of two chromosomes and one plasmid (designated pDW0551), was sequenced. The larger chromosome (chromosome_1) is approximately 2.67 Mb in size, encoding 2,787 open reading frames (ORFs) with an average length of 847 bp, and the other (chromosome_2) is approximately 1.97 Mb in length, encoding 2,034 ORFs with an average length of 871 bp (**Figure 1**). The plasmid is 221.64 kb in size and encodes 250 ORFs (**Table 3**). Species identification analysis of DW0551 revealed that it shared the highest genome-wide ANI (97.83%) with the type strain *Brucella intermedia* 34576_H01 (GenBank assembly accession: GCA_900454225.1) in the NCBI nucleotide database, and 16S rRNA gene homology analysis revealed that the 16S rRNA gene of DW0551 shared the highest similarity (99.66%) with that of *Brucella intermedia* O. intermedium_CIP_105838 (GCA_012103055.1). According to the criteria for classifying a bacterium as a certain species (a threshold of ≥ 95% ANI was set to classify a bacterium as a certain species) (Richter and Rosselló-Móra, 2009), the isolate DW0551 belonged to the species *Brucella intermedia* and was thus designated *Brucella intermedia* DW0551.

**Table 3:** General features of the DW0551 genome.

	Chromosome_1	Chromosome_2	pDW0551
Size (bp)	2,670,871	1,968,216	221,642
GC content (%)	58.18	57.24	53.46
ORFs	2,787	2,034	250
Known proteins	2,084 (76.79%)	1,450 (72.54%)	117 (46.80%)
Hypothetical proteins	630 (23.21%)	549 (27.46%)	133 (53.20%)
Protein coding (%)	97.38	98.28	96.15
Average ORF length (bp)	847	871	829.8
Average protein length (aa)	286.8	297.1	265.3
tRNA	40	17	0
rRNA	(16S-23S-5S) × 1	(16S-23S-5S) × 2	0

A total of 59 *Brucella intermedia* genomes were present in the NCBI genome database, the genome sizes of which ranged from 3.94 Mb (GCA_001637305.1) to 5.39 Mb (GCA_028621395.1). Only four strains had complete genomes, namely, *B. intermedia* ZJ499 (GCA_014495725.1), *B. intermedia* SG. G2 (GCA_025490555.1), *B. intermedia* ZL (GCA_029834515.1) and *B. intermedia* TSBOI (GCA_029855085.1). The remaining genomes were incomplete draft genomes. The four complete genomes each contained two chromosomes, and only *B. intermedia* SG. G2 had a plasmid, which was 176.21 kb in length, approximately 45 kb smaller than the plasmid pDW0551 in this study. When searching for similar plasmid sequences in the NCBI nucleotide database, no sequence that shared an identity of more than 10% with pDW0551 was found.

The results of antibiotic susceptibility testing revealed that DW0551 had high MIC levels of ≥ 16 µg/mL to 81.40% (35/43) of the antimicrobial agents tested. The MICs for aminoglycosides were especially high. Among the 10 aminoglycosides tested, except for a relatively low MIC value of 16 µg/mL for neomycin, DW0551 exhibited high MICs for the other 9 antimicrobial agents, with MIC ≥ 512 µg/mL for sisomicin and ≥ 64 µg/mL for netilmicin (**Table 2**). Compared to the recombinant carrying *aac(6’)-Iaq* DH5α (pMD19-T-*aac(6’)-Iaq*), the wild strain DW0551 showed higher MIC levels to aminoglycosides tested. This variation may be due to the presence of other resistance mechanisms, such as efflux pumps, modifying enzymes, target bypass, different intrinsic resistance mechanisms between different bacterial species and so on.

Considering the resistance genotype of the genome, a total of 106 drug resistance-related genes that shared amino acid identities ≥ 30% with functionally characterized resistance genes were predicted (**Supplementary Table S2**). Among them, 8 were putative aminoglycoside resistance genes encoding aminoglycoside-modifying enzymes, of which both *aac(6’)-Il* (Bunny et al., 1995) and *ant(2’’)-Ia* (Cox et al., 2015) shared amino acid identities of 100% with the functionally characterized resistance genes, while the remaining six (including the novel resistance gene *aac(6’)-Iaq* of this work) shared amino acid identities ranging from 44.1% to 73.2% with the functionally characterized genes.

### Functional determination and molecular characterization of the novel aminoglycoside acetyltransferase gene *aac(6’)-Iaq*

To determine the drug resistance function of the *aac(6’)-Iaq* gene, the ORF of the *aac(6’)-Iaq* gene with its promoter region was cloned (the *aac(6’)-Iaq* gene and its flanking regions, including the proposed -10 and -35 regions of the promoter, are shown in **Supplementary Figure S1**), and the recombinant strain DH5α(pMD19-T-*aac(6’)-Iaq*) exhibited resistance to numerous aminoglycosides, including ribostamycin, netilmicin, sisomicin, tobramycin, gentamicin, kanamycin, and amikacin, with 128-, 64-, 64-, 32-, 8-, 8-, and 8-fold higher MIC levels, respectively, than that of the control strain DH5α(pMD19-T) (**Table 2**). However, the MICs of spectinomycin and streptomycin did not vary.

**Figure 2:**
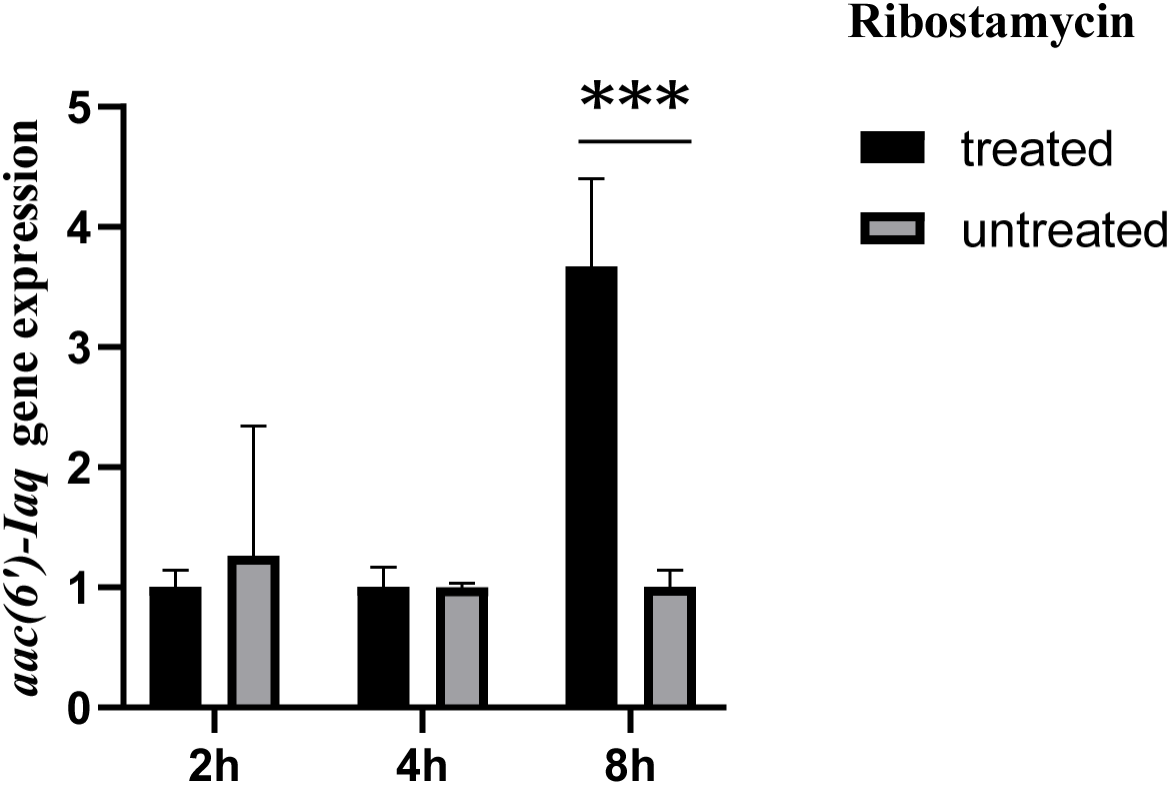
Comparison of the expression levels of the *aac(6’)-Iaq* gene in the recombinant strains treated with or without 1024 µg/mL ribostamycin. The bars indicate the means ± standard errors, and the experiments were conducted in triplicate. A statistically significant difference was observed between the treated and untreated groups at 8 h. “***”: Significant difference, *P* < 0.05.

**Figure 3:**
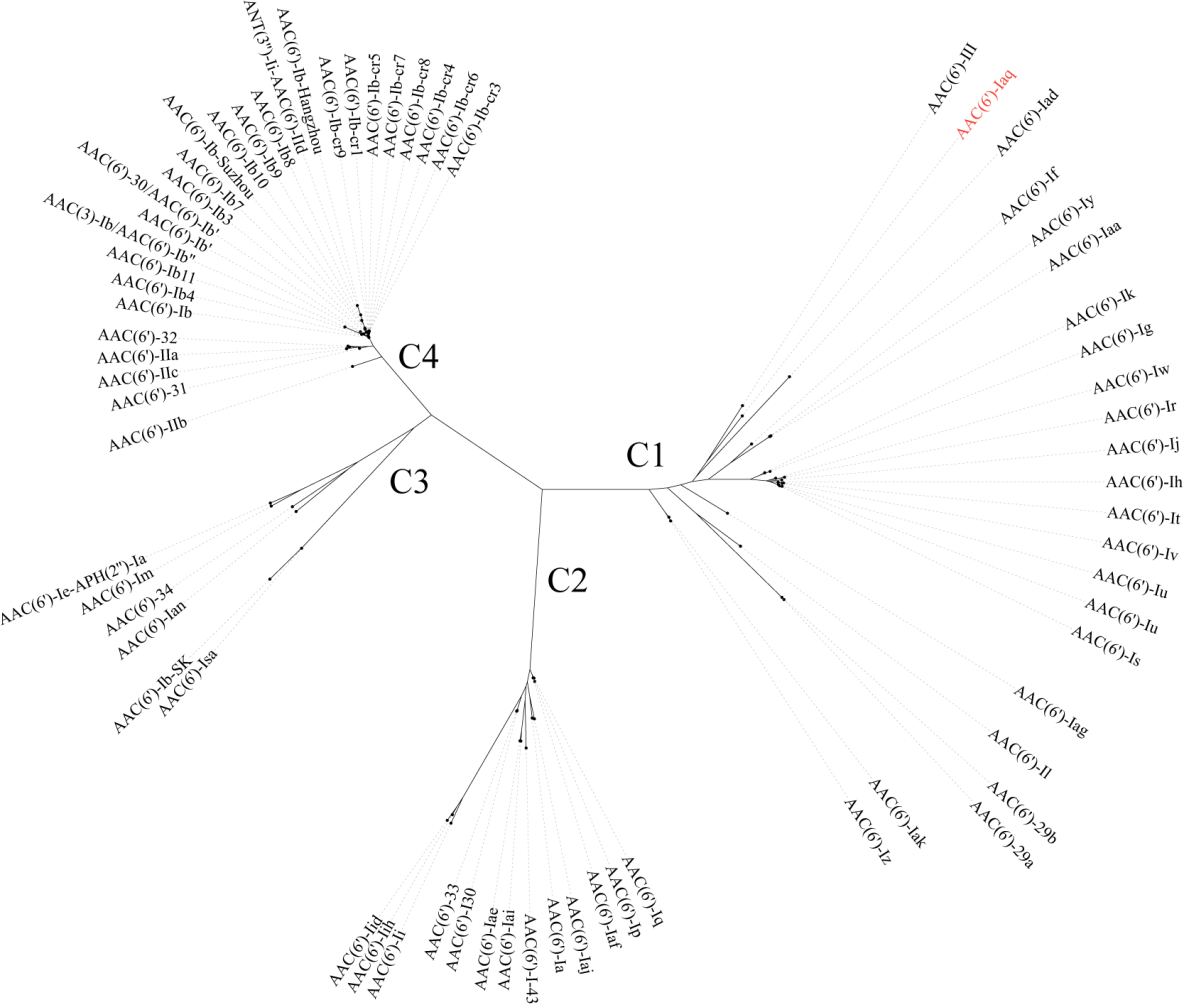
A phylogenetic tree showing the relationship of AAC(6’)-Iaq with other functionally characterized AAC(6’)s; C1-C4 refer to clusters 1-4. AAC(6’)-Iaq is highlighted in red. The accession numbers of these genes are listed in **Supplementary Table S3**.

The *aac(6’)-Iaq* gene encoded in the chromosome 1 (**Figure 1**) is 447 bp in length and encodes a protein of 148 amino acids with a theoretical pI value of 4.64. To investigate the possible induction of *aac(6’)-Iaq* expression by antibiotics, we performed RT-qPCR experiments to compare the relative expression levels of the genes in the presence and absence of antibiotics. After induction by ribostamycin for 8 h, the expression of the *aac(6’)-Iaq* gene in the ribostamycin-treated group increased approximately 3-fold in comparison to that in the control group (P < 0.05) (**Figure 2**). When analyzing the relationship of *aac(6’)-Iaq* with functionally characterized proteins, a total of 4 functionally characterized resistance genes (all *aac(6’)-I* genes) with aa identities greater than 50% were found in the public antibiotic resistance gene database CARD. These genes were *aac(6’)-If* (Ploy et al., 1994), *aac(6’)-Iaa* (Salipante and Hall, 2003), *aac(6’)-Iy* (Magnet et al., 1999) and *aac(6’)-Ic* (Shaw et al., 1992), and the proteins they encoded shared amino acid identities of 52.63%, 52.05%, 51.37% and 50.74%, respectively, with that encoded by *aac(6’)-Iaq*. Evolutionary analysis of all the members of the functionally characterized AAC(6’) family revealed that they were roughly clustered into 4 clusters (C1-C4) (**Figure 3**; **Supplementary Table S3**). The AAC(6’) genes that shared higher identities with *aac(6’)-Iaq* were clustered together.

To compare the resistance spectra of strains harboring different *aac(6’)* genes, the resistance phenotypes of 13 *aac(6’)* genes from different evolutionary clusters were analyzed; four of these genes were most closely related to *aac(6’)-Iaq* (no resistance profile was available for *aac(6’)-If*) in cluster 1, and the remaining 9 *aac(6’)* genes were randomly selected from the other 3 clusters (**Supplementary Table S4**). The novel aminoglycoside resistance gene *aac(6’)-Iaq* conferred resistance to all seven aminoglycosides detected; however, for the other 13 *aac(6’)* genes, among the 4 to 7 antimicrobial agents tested, each showed susceptibility to 1 or 2 antirobial agents. Among the three antimicrobial agents tested, all the genes conferred resistance to tobramycin, and most (12/14) conferred resistance to amikacin; however, only a small portion (4/14) of the genes conferred resistance to gentamicin. All of the genes tested conferred resistance to the antimicrobial molecules tested, except for *aac(6’)-Iaf* (Kitao et al., 2009) and *aac(6’)-Isa* (Hamano et al., 2004), both of which showed susceptibility to sisomicin. The *aac(6’)* genes exhibited different resistance spectra even though they were phylogenetically closely related.

**Figure 4:**
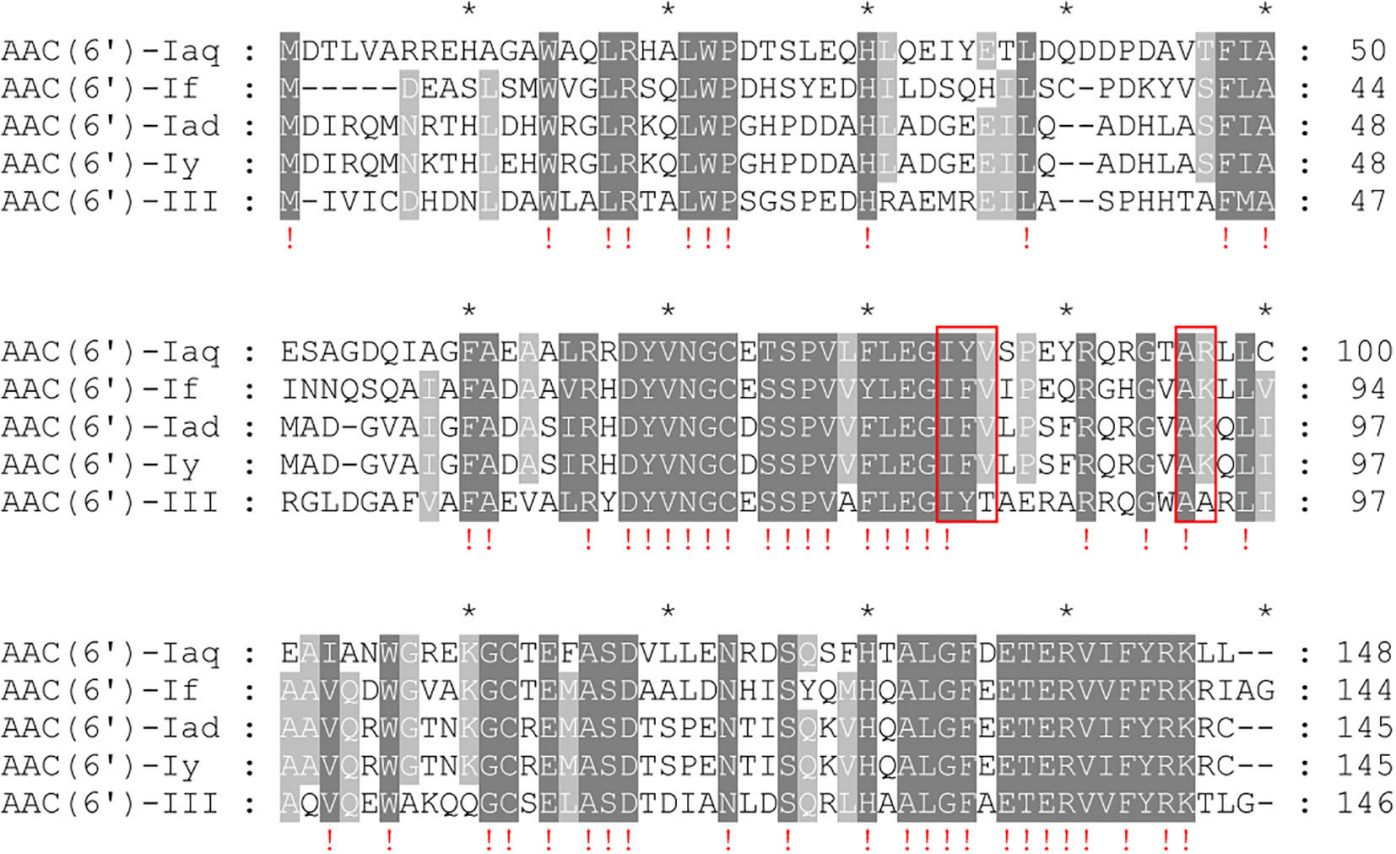
Multiple sequence alignment of AAC(6’)-Iaq with other close relatives of the C1 cluster. The numbers on the right represent the corresponding amino acid sequence length. Exclamation marks indicate fully conserved residues; gaps are represented using hyphens. The red frames represent the residues of the conserved protein domain family NAT_SF. “*” is a location marker. Dark gray indicates conserved sites, and light gray indicates relatively conserved sites.

When analyzing the essential functional residues of the protein, multiple sequence alignment of AAC(6’)-Iaq with the functionally characterized proteins of the AAC(6’)-I proteins was performed (Marchler-Bauer et al., 2017). The coenzyme A chemical binding pocket of the conserved protein domain family NAT_SF (PSSM-Id: 173926), consisting of 5 residues (I84, Y85, V86, A96 and R97) (Rojas et al., 1999), was present in AAC(6’)-Iaq (I^84^-Y^85^-V^86^-X-X-X-X-X-X-X-X-X-A^96^-R^97^) (**Figure 4**). The conserved protein domain family NAT_SF belongs to the cl17182 superfamily (domain architecture ID 10456837, a family of proteins containing various enzymes with catalytic acyl-transfer substrates) (Wybenga-Groot et al., 1999).

### *aac(6’)-Iaq* showed higher affinity and catalytic efficiency for netilmicin than the other related proteins

The results of the acetyltransferase activity and enzyme kinetic parameter analyses of AAC(6’)-Iaq demonstrated that, consistent with the MIC results, AAC(6’)-Iaq was able to acetylate ribostamycin, netilmicin, sisomicin, kanamycin and amikacin. No acetyltransferase activity could be detected for streptomycin. According to the Michaelis-Menten constant, this enzyme had the highest affinities and catalytic efficiencies for netilmicin [*K*_m_ of 3.83 ± 0.34 µM and *k*_cat_/*K*_m_ of (9.08 ± 1.83) × 10^4^ M^-1^/s^-1^] and sisomicin [*K*_m_ of 4.33 ± 1.32 µM and *k*_cat_/*K*_m_ of (6.13 ± 2.26) × 10^4^ M^-1^/s^-1^] (**Table 4**).

**Table 4:** Kinetic parameters of AAC(6’)-Iaq.

Substrate	*K*_cat_ (s^-1^)	*K*_m_ (µM)	*k*_cat_/*K*_m_ (M^-1^/s^-1^)
sisomicin	0.25 ± 0.03	4.33 ± 1.32	(6.13 ± 2.26) × 10^4^
ribostamycin	0.17 ± 0.004	10.05 ± 2.45	(1.83 ± 0.45) × 10^4^
netilmicin	0.34 ± 0.04	3.83 ± 0.34	(9.08 ± 1.83) × 10^4^
kanamycin	0.49 ± 0.08	13.34 ± 9.07	(4.64 ± 2.25) × 10^4^
amikacin	0.46 ± 0.08	14.08 ± 0.76	(3.26 ± 0.72) × 10^4^
streptomycin	NA^a^	NA^a^	NA^a^

a NA, no activity observed.

In addition to streptomycin, which has a hydroxyl group at the 6’-position, AAC(6’)-Iaq exhibited acetyltransferase activity toward five other aminoglycosides harboring an amino group at the 6’ position, and this regiospecific acetyltransferase transfer is consistent with previous findings (Magnet et al., 2001). Among these aminoglycoside substrates, amikacin and kanamycin were poor substrates, exhibiting lower affinities (with *K*_m_ values of 14.08 ± 0.76 µM and 13.34 ± 9.07 µM, respectively); this difference is thought to be related to the fact that the substituent at position 1 of ring I of amikacin and kanamycin is a hydroxyl group rather than an amino group (Tada et al., 2016).

AAC(6’)-Iaq exhibits a higher affinity and a higher catalytic efficiency for netilmicin than several other AAC(6’) enzymes reported previously. The *K*_m_ values for netilmicin of AAC(6’)-Ial, AAC(6)-Iy, and AAC(6’)-Ic were 23 ± 6, 8 ± 1 and 20 ± 5 µM, respectively (Magnet et al., 2001; Tada et al., 2016), while the *k*_cat_/*K*_m_ values for netilmicin of AAC(6’)-Ial, AAC(6’)-Iap and AAC(6’)-III [AAC(6’)-Ic] were 4.1×10^4^ M^-1^/s^-1^, 2.2 × 10^4^ M^-1^/s^-1^, and 2.9×10^4^ M^-1^/s^-1^, respectively (Kim et al., 2007; Tada et al., 2016); however, AAC(6’)-Ib (Vetting et al., 2008) had a much greater catalytic efficiency for netilmicin than the other AAC(6’)-I proteins, showing a *k*_cat_/*K*_m_ value of (2.0 ± 0.5) × 10^6^ M^-1^/s^-1^.

### The *aac(6’)-Iaq* gene related sequences conserved in *Brucella* species

**Figure 5:**
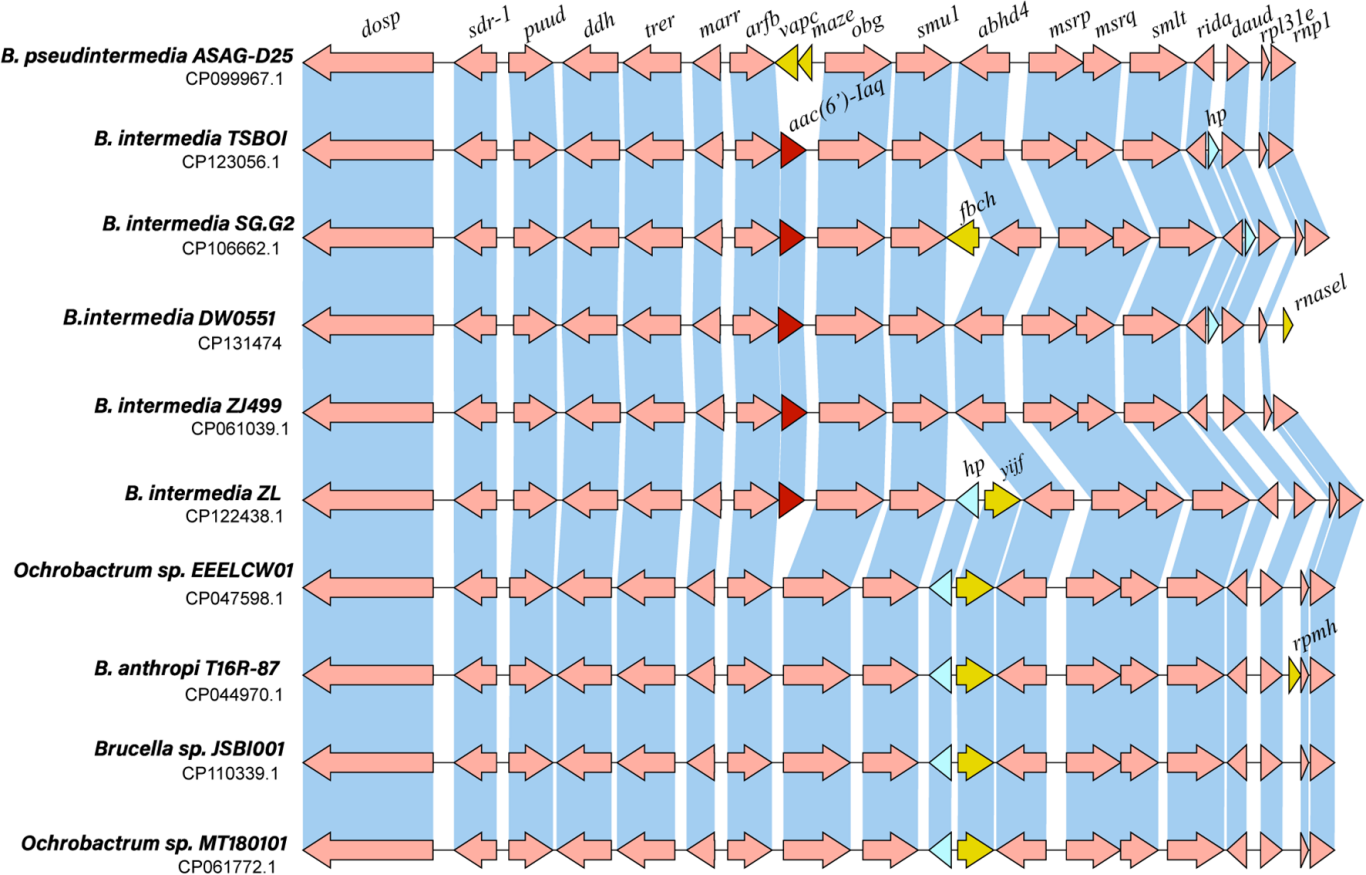
Genetic context of *aac(6’)-Iaq* and other related sequences. Regions with ≥ 80% amino acid identity are in blue. hp: hypothetical protein. The *aac(6’)-Iaq* and *aac(6’)-Iaq*-like genes (similarity > 95.0%) are shown in red. The genes specific to each sequence are shown in yellow, and the hypothetical protein-encoding genes are shown in blue.

To analyze the genetic context of the *aac(6’)-Iaq-*encoding region, a sequence approximately 20 kb in length with *aac(6’)-Iaq* at the center was used as a query to search the nonredundant nucleotide database of the NCBI. A total of 18 sequences with > 80% nucleotide identity were retrieved, and all of them were from the genus *Brucella*/*Ochrobactrum*. Of these 18 sequences, the four sequences with the highest identities (> 96.0%) were from *Brucella intermedia* (CP123056.1, identity 98.29%, CP061039.1, identity 97.20%, CP122438.1, identity 97.04%, and CP106662.1, identity 96.98%), and all of them contained an *aac(6’)-Iaq*-like gene (an *aac(6’)-Iaq-*like gene is a gene other than *aac(6’)-Iaq* according to the public database and the protein it encoded shares aa identity of ≥ 80% with AAC(6’)-Iaq). The remaining 14 sequences showed identities ranging from 82.24% to 85.64% and were all free of an *aac(6’)-Iaq*-like gene. Comparative structural analysis of 10 sequences, including four sequences that shared identities higher than 96.0% and five sequences that shared lower identities with the sequence of interest in this work, was performed, and the results revealed that they had similar genetic contexts in terms of gene content and gene order. Except for the *aac(6’)-Iaq*-like gene, at most one or two predicted ORFs (such as *vapC*, *mazE*, *fbcH*, *rnasel, yijF, rpmH* or a hypothetical protein-encoding gene) differed between them (**Figure 5**). No MGE was found within the 20 kb sequences. However, the mechanism underlying its appearance in *Brucella intermedia* chromosomes remains to be further studied.

To analyze the distribution of the *aac(6’)-Iaq*(-like) genes, the aa sequence of AAC(6’)-Iaq was used as a query to search the NCBI nonredundant nucleotide database, and approximately one hundred sequences with amino acid similarities greater than 50.0% were retrieved. Of these sequences, only four (mentioned above) had similarities > 95.0% and they were from the same species as AAC(6’)-Iaq, *Brucella intermedia.* As mentioned above, there are four complete *Brucella intermedia* genomes in the NCBI genome database, and the sequences carrying the AAC(6’)-Iaq(-like) proteins are from them, respectively: CP106662.1 (identity 97.3% chromosome of SG.G2), isolated from houseplant; CP122438.1 (identity 96.62%, chromosome of ZL), isolated from environment; CP061039.1 (identity 96.62%, chromosome of ZJ499), isolated from human sputum; CP123056.1 (identity 95.95%, chromosome of TSBOI), isolated from soil, and all the others had similarities < 60% with AAC(6’)-Iaq; these similar sequences were from bacteria of different families, such as one (CP120373.1, identity 54.60%) from *Ensifer garamanticus* LMG 24692, or from different classes, such as one (CP116005.1, identity 59.59%) from *Sphingosinicella microcystinivorans* DMF-3. The genetic context of these sequences was entirely different from that of the sequence in this work (*Brucella intermedia* DW0551). To analyze the evolutionary relationships between these genes, additional *aac(6’)-Iaq*-like genes with higher identities would be included.

## Conclusion

In this study, a novel aminoglycoside resistance gene, designated *aac(6’)-Iaq*, encoded on the chromosome of a *Brucella intermedia* isolate from goose feces, was identified. AAC(6’)-Iaq shares < 60% amino acid identity with all functionally characterized aminoglycoside resistance gene encoded proteins and shows resistance to several aminoglycosides including ribostamycin, netilmicin, sisomicin, tobramycin, gentamicin, kanamycin, and amikacin. AAC(6’)-Iaq exhibited acetylation activity toward the aminoglycoside substrates analyzed. The discovery of novel resistance mechanisms in animal bacteria might also aid in the treatment of microbial infections in animals and humans.

## Data Availability

The datasets presented in this study can be found in online repositories. The names of the repository/repositories and accession number(s) can be found in the article/**Supplementary Material**.

## Supplementary material

Supplementary Figure 1 The *aac(6’)-Iaq* gene and its flanking regions. The underlined regions are the -10 and -35 regions of the proposed promoter. The *aac(6’)-Iaq* gene is shaded gray.

Supplementary Table 1 Primers used in this study.

Supplementary Table 2 106 resistance genes predicted from the DW0551 genome.

Supplementary Table 3 Sequences used to reconstruct the phylogenetic tree.

Supplementary Table 4 Resistance phenotypes conferred by *aac(6’)* genes belonging to different phylogenetic clusters.
