# Supplementary figures and images for: aac(6’)-Iaq, a novel aminoglycoside acetyltransferase gene identified from an animal isolate Brucella intermedia DW0551
Source: Front Cell Infect Microbiol. 2025 Mar 11;15:1551240. doi: 10.3389/fcimb.2025.1551240 (PMC11932996; doi:10.3389/fcimb.2025.1551240)

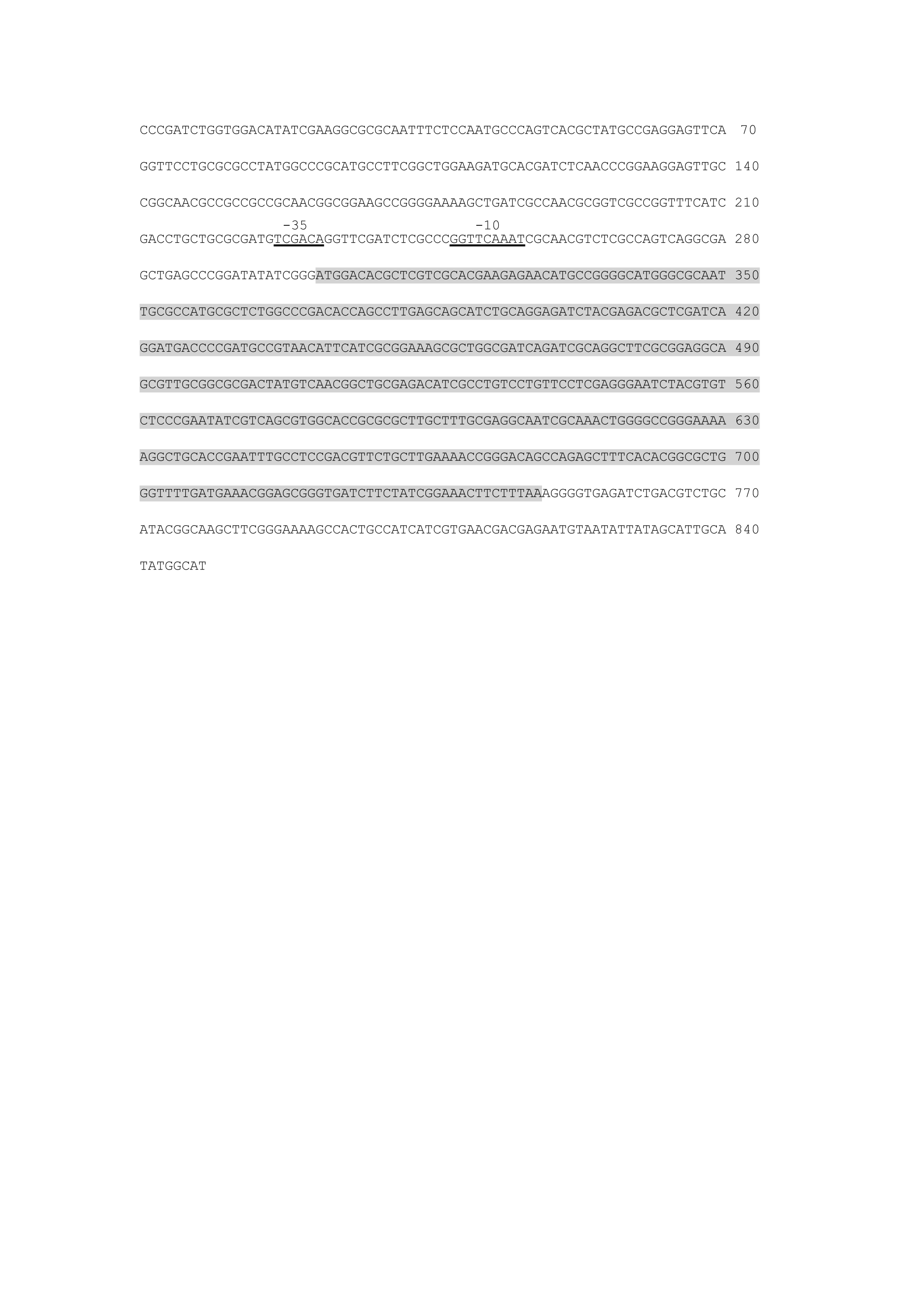

Supplement: Supplementary Figure 1 — The aac(6’)-Iaq gene and its flanking regions. The underlined regions are the -10 and -35 regions of the proposed promoter. The aac(6’)-Iaq gene is shaded gray. [file Image1.tif]
