# Supplementary material for: aac(6’)-Iaq, a novel aminoglycoside acetyltransferase gene identified from an animal isolate Brucella intermedia DW0551
Source: Front Cell Infect Microbiol. 2025 Mar 11;15:1551240. doi: 10.3389/fcimb.2025.1551240 (PMC11932996; doi:10.3389/fcimb.2025.1551240)
Supplement: Supplementary Table 1 — Primers used in this study. [file Table1.docx]

Table S1 Primers used in this study.

| Primer^a^ | Sequence (5’ → 3’) | Vector | Restriction endonuclease |
| --- | --- | --- | --- |
| F-551-clo | CGGATCCTTCATCGACCTGCTGCGCGAT | pMD19-T | BamHI |
| R-551-clo | CGAATTCCTTGCCGTATGCAGACGTCAGA-TCT | pMD19-T | EcoRI |
| F-551-exp | CCAGCTTGGGGTACCCTGGTGCCGCGCG-GCAGCATGGACACGCTCGTCGCACGAAGAG | pCold I | HindIII |
| R-551-exp | GGGGTACCCCAAGCTTCGAAGCTTGCCG-TATGCAGACGTCAGATCT | pCold I | KpnI |

^a^Primers ending with “clo” were used to clone the ORF of the *aac(6’)-Iaq* gene and its promoter region; primers ending with “exp” were used to clone the ORF of the *aac(6’)-Iaq* gene. Restriction enzyme sites are underlined.
