# Supplementary material for: aac(6’)-Iaq, a novel aminoglycoside acetyltransferase gene identified from an animal isolate Brucella intermedia DW0551
Source: Front Cell Infect Microbiol. 2025 Mar 11;15:1551240. doi: 10.3389/fcimb.2025.1551240 (PMC11932996; doi:10.3389/fcimb.2025.1551240)
Supplement: Supplementary Table 2 — 106 resistance genes predicted from the DW0551 genome. [file Table2.docx]

Table S2 106 resistance genes predicted from the DW0551 genome.

| No. | Gene | Identity (%) | Accession No. | No. | Gene | Identity (%) | Accession No. |
| --- | --- | --- | --- | --- | --- | --- | --- |
| 1 | *erm(37)* | 30.19 | 3000392 | 54 | *tet(T)* | 34.81 | 3003378 |
| 2 | *nmcR* | 30.22 | 3003665 | 55 | s*ta* | 35.05 | 3000193 |
| 3 | *vanH* | 30.48 | 3002945 | 56 | *basS* | 35.19 | 3004699 |
| 4 | *eFTu* | 30.57 | 3003361 | 57 | *rphB* | 35.23 | 3003583 |
| 5 | *soxS* | 30.69 | 3003511 | 58 | *tlrC* | 35.68 | 3003992 |
| 6 | *basS* | 30.72 | 3003583 | 59 | *acrR* | 35.94 | 3002827 |
| 7 | *tsnR* | 30.77 | 3003060 | 60 | *soxR* | 36.21 | 3003807 |
| 8 | *inhA* | 30.8 | 3003393 | 61 | *aac(6')-Ib11* | 36.36 | 3003381 |
| 9 | *vanR* | 30.86 | 3002921 | 62 | *erm(37)* | 36.36 | 3002582 |
| 10 | *vanH* | 31.25 | 3002945 | 63 | *nmcR* | 36.59 | 3000392 |
| 11 | *vatB* | 31.28 | 3002841 | 64 | *cmlv* | 36.6 | 3003665 |
| 12 | *iri* | 31.33 | 3002884 | 65 | *eptA* | 36.72 | 3002700 |
| 13 | *uhpA* | 31.41 | 3003893 | 66 | *sta* | 37.31 | 3003576 |
| 14 | *tsnR* | 31.47 | 3003060 | 67 | *gyrA* | 37.8 | 3004699 |
| 15 | *tetR* | 31.48 | 3003479 | 68 | *vanR* | 38.02 | 3003995 |
| 16 | *cprR* | 31.58 | 3005063 | 69 | *vanG* | 38.13 | 3003728 |
| 17 | *acrR* | 31.65 | 3003807 | 70 | *kasA* | 38.28 | 3002909 |
| 18 | *arnA* | 31.67 | 3002985 | 71 | *ompA* | 38.46 | 3003463 |
| 19 | *mupB* | 31.82 | 3000510 | 72 | *cprR* | 39.09 | 3005044 |
| 20 | *pbp1* | 31.82 | 3007060 | 73 | *llmA* | 40 | 3005063 |
| 21 | *l1_BLA* | 31.87 | 3000582 | 74 | *kasA* | 40.43 | 3003982 |
| 22 | *mexR* | 31.91 | 3000506 | 75 | *nmcR* | 42.31 | 3003463 |
| 23 | *vanR* | 31.93 | 3002925 | 76 | *gyrB* | 42.53 | 3003665 |
| 24 | *uhpA* | 31.94 | 3003893 | 77 | *lmrC* | 42.75 | 3004562 |
| 25 | *satA* | 32.14 | 3005045 | 78 | *edeQ* | 42.76 | 3002881 |
| 26 | *ugd* | 32.23 | 3003577 | 79 | *bacA* | 43.23 | 3004063 |
| 27 | *acrR* | 32.26 | 3003807 | 80 | *catB2* | 43.79 | 3002986 |
| 28 | *soxS* | 32.26 | 3003511 | 81 | *aph(6)-Id* | 44.09 | 3002675 |
| 29 | *vanH* | 32.39 | 3007188 | 82 | *catB10* | 44.23 | 3002660 |
| 30 | *nmcR* | 32.55 | 3003665 | 83 | *sul4* | 44.32 | 3003110 |
| 31 | *acrR* | 32.56 | 3003807 | 84 | *basR* | 44.91 | 3004361 |
| 32 | *uhpA* | 32.65 | 3003893 | 85 | *tet(Q)* | 45.45 | 3003582 |
| 33 | *bcrC* | 32.81 | 3003250 | 86 | *nmcR* | 46.18 | 3000191 |
| 34 | *pBP2* | 32.83 | 3004832 | 87 | *rpoB* | 47.12 | 3003665 |
| 35 | *sta* | 32.84 | 3004699 | 88 | *dfrA26* | 47.56 | 3007051 |
| 36 | *carA* | 32.97 | 3002817 | 89 | *aph(3')-IIb* | 48.25 | 3002857 |
| 37 | *pmrF* | 33.05 | 3003578 | 90 | *gyrA* | 48.47 | 3002645 |
| 38 | *ompA* | 33.08 | 3005044 | 91 | *ant(3'')-Ib* | 48.91 | 3003995 |
| 39 | *nmcR* | 33.1 | 3003665 | 92 | *tetR* | 49.25 | 3005062 |
| 40 | *pbp1* | 33.11 | 3007060 | 93 | *murA* | 49.3 | 3003479 |
| 41 | *vanH* | 33.2 | 3007188 | 94 | *aac(6')-If* | 52.05 | 3003776 |
| 42 | *vanH* | 33.2 | 3002942 | 95 | *fabI* | 52.12 | 3002571 |
| 43 | *soxS* | 33.33 | 3003511 | 96 | *fusA* | 58.17 | 3004045 |
| 44 | *sta* | 33.7 | 3004699 | 97 | *soxR* | 58.74 | 3003735 |
| 45 | *otr(A)* | 33.83 | 3002891 | 98 | *thyA* | 60.61 | 3004107 |
| 46 | *cfrC* | 34.01 | 3004146 | 99 | *eFTu* | 74.18 | 3004153 |
| 47 | *vanR* | 34.07 | 3002929 | 100 | *eFTu* | 75.56 | 3003369 |
| 48 | *soxS* | 34.12 | 3003511 | 101 | *gyrA* | 77.92 | 3003369 |
| 49 | *pbp1* | 34.16 | 3007060 | 102 | *och-6* | 84.62 | 3003302 |
| 50 | *arnA* | 34.27 | 3002985 | 103 | *mprF* | 89.23 | 3002519 |
| 51 | *sta* | 34.41 | 3004699 | 104 | *aac(6')-Il* | 100 | 3003772 |
| 52 | *optrA* | 34.42 | 3003746 | 105 | *ant(2'')-Ia* | 100 | 3004635 |
| 53 | *marR* | 34.57 | 3000392 | 106 | *sul1* | 100 | 3000230 |
