# Supplementary material for: aac(6’)-Iaq, a novel aminoglycoside acetyltransferase gene identified from an animal isolate Brucella intermedia DW0551
Source: Front Cell Infect Microbiol. 2025 Mar 11;15:1551240. doi: 10.3389/fcimb.2025.1551240 (PMC11932996; doi:10.3389/fcimb.2025.1551240)
Supplement: Supplementary Table 3 — Sequences used to reconstruct the phylogenetic tree. [file Table3.docx]

Table S3 Sequences used to reconstruct the phylogenetic tree.

| Protein | ARO accession No. | Protein accession No. | DNA accession No. |
| --- | --- | --- | --- |
| AAC(3)-Ib/AAC(6')-Ib'' | ARO:3002600 | AAL82588.1 | AF355189.1 |
| AAC(6')-29a | ARO:3002583 | WP_064190968.1 | NG_048575.1 |
| AAC(6')-29b | ARO:3002584 | WP_064190969.1 | NG_048576.1 |
| AAC(6')-30/AAC(6')-Ib' | ARO:3002599 | CAE48335.2 | AJ584652.2 |
| AAC(6')-31 | ARO:3002585 | CAK55557.1 | AM283489.1 |
| AAC(6')-32 | ARO:3002586 | ABR10839.1 | EF614235.1 |
| AAC(6')-33 | ARO:3002587 | AEZ05106.1 | JN596280.1 |
| AAC(6')-34 | ARO:3003989 | APB03223.1 | KX531053.1 |
| AAC(6')-I30 | ARO:3002588 | AAP43642.1 | AY289608.1 |
| AAC(6')-Ia | ARO:3002545 | AAA98298.1 | M18967.1 |
| AAC(6')-Iaa | ARO:3002571 | AAL20537.1 | AE006468.2 |
| AAC(6')-Iad | ARO:3002572 | BAD12078.1 | AB119105.1 |
| AAC(6')-Iae | ARO:3002573 | BAD14386.1 | AB104852.1 |
| AAC(6')-Iaf | ARO:3002574 | BAH66386.1 | AB462903.1 |
| AAC(6')-Iag | ARO:3004638 | BAN78519.1 | AB472901.2 |
| AAC(6')-Iai | ARO:3002575 | ACI28880.1 | EU886977.1 |
| AAC(6')-Iaj | ARO:3003677 | BAM46120.1 | AB709942.1 |
| AAC(6')-Iak | ARO:3003199 | BAO21229.1 | AB894482.1 |
| AAC(6')-Ian | ARO:3003200 | BAQ22025.1 | AP014611.1 |
| AAC(6')-Ib | ARO:3002546 | AFJ11384.1 | JQ808129.1 |
| AAC(6')-Ib' | ARO:3003676 | AAT74613.1 | AY660529.1 |
| AAC(6')-Ib10 | ARO:3002581 | AAC46343.1 | U59183.1 |
| AAC(6')-Ib11 | ARO:3002582 | AAN41403.1 | AY136758.1 |
| AAC(6')-Ib3 | ARO:3002576 | ACS44715.1 | FJ854362.1 |
| AAC(6')-Ib4 | ARO:3002577 | AAL38577.1 | AF445082.1 |
| AAC(6')-Ib7 | ARO:3002578 | AKN19287.1 | KR091911.1 |
| AAC(6')-Ib8 | ARO:3002579 | AIK02012.1 | KF998105.1 |
| AAC(6')-Ib9 | ARO:3002580 | AAD02244.1 | AF043381.1 |
| AAC(6')-Ib-cr1 | ARO:3002547 | WP_071846215.1 | NG_052213.1 |
| AAC(6')-Ib-cr3 | ARO:3005112 | ADY02579.1 | HQ170516.1 |
| AAC(6')-Ib-cr4 | ARO:3005114 | KJX28101.1 | JZKY01000061.1 |
| AAC(6')-Ib-cr5 | ARO:3005115 | ABX24471.1 | EU161636.1 |
| AAC(6')-Ib-cr6 | ARO:3005116 | ACD56150.1 | EU675686.2 |
| AAC(6')-Ib-cr7 | ARO:3005117 | NON98701.1 | JABGAB010000032.1 |
| AAC(6')-Ib-cr8 | ARO:3005118 | AZK52946.1 | CP034250.1 |
| AAC(6')-Ib-cr9 | ARO:3005119 | AYD68572.1 | MH569711.1 |
| AAC(6')-Ib-Hangzhou | ARO:3002592 | ACL37342.1 | FJ503047.1 |
| AAC(6')-Ib-SK | ARO:3002593 | BAD11815.1 | AB164230.1 |
| AAC(6')-Ib-Suzhou | ARO:3002591 | ABU55430.1 | EU085533.1 |
| AAC(6')-Ic[AAC(6’)-III] | ARO:3002549 | AAA26549.1 | M94066.1 |
| AAC(6')-Ie-APH(2'')-Ia | ARO:3002597 | AAA88548.1 | GU565967.1 |
| AAC(6')-If | ARO:3002553 | CAA39038.1 | X55353.1 |
| AAC(6')-Ig | ARO:3002554 | AAA21889.1 | L09246.1 |
| AAC(6')-Ih | ARO:3002555 | AAC41391.1 | L29044.1 |
| AAC(6')-Ii | ARO:3002556 | AAB63533.1 | L12710.1 |
| AAC(6')-IIa | ARO:3002594 | AAM92464.1 | AY123251.1 |
| AAC(6')-IIb | ARO:3002595 | AAA25680.1 | L06163.1 |
| AAC(6')-IIc | ARO:3002596 | AAD46626.1 | AF162771.1 |
| AAC(6')-Iid | ARO:3002589 | CAE50925.1 | AJ584700.2 |
| AAC(6')-Iih | ARO:3002590 | CAE50926.1 | AJ584701.2 |
| AAC(6')-Ij | ARO:3002557 | AAC41392.1 | L29045.1 |
| AAC(6')-Ik | ARO:3002558 | AAA87229.1 | L29510.1 |
| AAC(6')-Il | ARO:3004635 | AAA90937.1 | U13880.2 |
| AAC(6')-Im | ARO:3004629 | AAK63041.1 | AF337947.1 |
| AAC(6')-Ip | ARO:3002559 | CAA91010.1 | Z54241.1 |
| AAC(6')-Iq | ARO:3002560 | AAC25500.1 | AF047556.1 |
| AAC(6')-Ir | ARO:3002561 | AAD03490.1 | AF031326.1 |
| AAC(6')-Is | ARO:3002562 | AAD03491.1 | AF031327.1 |
| AAC(6')-Isa | ARO:3002563 | BAD10948.2 | AB116646.1 |
| AAC(6')-It | ARO:3002564 | AAD03492.1 | AF031328.1 |
| AAC(6')-Iu | ARO:3002565 | AAD03493.1 | AF031329.1 |
| AAC(6')-Iv | ARO:3002566 | AAD03494.1 | AF031330.1 |
| AAC(6')-Iw | ARO:3002567 | AAD03495.1 | AF031331.1 |
| AAC(6')-Ix | ARO:3002568 | AAD03496.1 | AF031332.1 |
| AAC(6')-Iy | ARO:3002569 | AAF03531.1 | AF144880.1 |
| AAC(6')-Iz | ARO:3002570 | AAD52985.1 | AF140221.1 |
| AAC(6')-I-43 | ARO:3004641 | AEA07977.1 | HQ247816.1 |
| ANT(3'')-Ii-AAC(6')-IId | ARO:3002598 | AAL51021.2 | AF453998.2 |
