# Supplementary material for: aac(6’)-Iaq, a novel aminoglycoside acetyltransferase gene identified from an animal isolate Brucella intermedia DW0551
Source: Front Cell Infect Microbiol. 2025 Mar 11;15:1551240. doi: 10.3389/fcimb.2025.1551240 (PMC11932996; doi:10.3389/fcimb.2025.1551240)
Supplement: Supplementary Table 4 — Resistance phenotypes conferred by aac(6’) genes belonging to different phylogenetic clusters. [file Table4.docx]

Table S4 Resistance phenotypes conferred by *aac(6’)* genes belonging to different phylogenetic clusters.

| Cluster | Gene | TOB | GEM | KAN | AMK | ISO | NET | SIS | RIB | Reference |
| --- | --- | --- | --- | --- | --- | --- | --- | --- | --- | --- |
| 1 | *aac(6')-Iaq* | R | R | R | R | NT | R | R | R | This study |
|  | *aac(6')-III* | R | S | NT | R | R | R | NT | NT | [(Shaw et al., 1992b)](#四十九) |
|  | *aac(6')-Iad* | R | S | R | R | R | NT | R | NT | [(Doi et al., 2004)](#十四) |
|  | *aac(6')-Iy* | R | S | NT | R | NT | R | NT | NT | (Magnet et al., 1999b) |
|  | *aac(6')-Iaa* | R | S | R | R | NT | NT | NT | NT | (Salipante and Hall, 2003) |
| 2 | *aac(6')-Iq* | R | S | R | R | R | R | NT | NT | [(Centrón and Roy, 1998)](#十) |
|  | *aac(6')-Ia* | R | R | NT | R | R | R | NT | NT | (Shaw et al., 1992a) |
|  | *aac(6')-Iaf* | R | S | R | R | R | R | S | NT | [(Kitao et al., 2009)](#二十七) |
| 3 | *aac(6')-Im* | R | S | R | R | NT | R | NT | NT | [(Chow et al., 2001)](#十一) |
|  | *aac(6')-Ian* | R | S | R | R | R | R | R | NT | [(Jin et al., 2015)](#二十三) |
|  | *aac(6')-Isa* | R | S | R | R | NT | NT | S | NT | [(Hamano et al., 2004)](#十八) |
| 4 | *aac(6')-IIa* | R | R | NT | S | NT | R | R | NT | [(Shaw et al., 1989)](#四十六) |
|  | *aac(6')-IIb* | R | R | R | S | NT | R | R | NT | [(Shaw et al., 1993)](#四十七) |
|  | *aac(6')-Ib* | R | S | NT | R | R | R | NT | NT | [(Shaw et al., 1992a)](#四十八) |

R, resistant; S, susceptible; NT, not tested; TOB, tobramycin; GEM, gentamicin; KAN, kanamycin; AMK, amikacin; ISO, isepamicin; NET, netilmicin; SIS, sisomicin; RIB, ribostamycin.
